# Teacher Feedback Practices, Student Feedback Motivation, and Feedback Behavior: How Are They Associated With Learning Outcomes?

**DOI:** 10.3389/fpsyg.2021.697045

**Published:** 2021-06-21

**Authors:** Zhengdong Gan, Zhujun An, Fulan Liu

**Affiliations:** ^1^Faculty of Education, University of Macau, Taipa, China; ^2^Foreign Languages College, Jiangxi Normal University, Nanchang, China

**Keywords:** teacher feedback, student feedback experience, learning outcomes, structural equation modeling, higher education

## Abstract

In spite of much recent theorizing about teacher provision of feedback, relatively fewer studies look at the dynamic relationships between teacher feedback practices, student feedback experience, and their learning outcomes in higher education settings. To fill this gap, this study looked at 308 university students' perceived teacher feedback practices and their feedback experiences in an English Studies course context at a key and non-key university, and explored how teacher feedback, student feedback motivation and feedback behavior were associated with students' course satisfaction and course exam performance. Results showed that students from the key university reported a higher level of teacher feedback use as well as student feedback motivation and behavior. Structural equation modeling (SEM) suggested that in the case of the non-key university, student feedback behavior significantly predicted course satisfaction and course exam results; teacher feedback also indirectly influenced course satisfaction and course exam results. In the case of the key university, while teacher feedback and student feedback behavior each had significant influence on course satisfaction, student feedback behavior showed no direct significant effect on course exam results, and teacher feedback also showed no significant indirect influence on course exam results.

## Introduction

It is recognized that learning is a matter not only of cognitive development but also of shared social practices (Walqui, 2006). Vygotsky (1978) claims that learning is a process of apprenticeship and internalization whereby skills and knowledge are transformed from the social into the cognitive plane. The concept of the zone of proximal development (ZPD) which is generally considered the core of Vygotsky's theory of learning is often interpreted as the distance between what a learner can do without help, and what they can do with support from someone with more knowledge or expertise. Applying this constructivist theory of learning to higher education, Toohey (2000) proposed a model of the learning process involving feedback intervention that functions as a kind of facilitating process enabling learner to master the new knowledge and use it in different and “real” situations. Toohey's model posits that the degree to which feedback facilitates learning depends on whether mistakes or misunderstandings are identified and whether any suggestions are given so that student work may improve. While feedback is a concept that has different meanings and interpretations, the dominant understanding of feedback in higher education is that it constitutes a teacher providing comments to a student in relation to his or her performance on a learning or assessment task (Carless, 2015). Although feedback is widely believed to contribute to student learning in schools (Black and Wiliam, 1998), some researchers (e.g., Higgins et al., 2002) raise doubts as to what extent this is reality in higher education, given institutional constraints and staff workloads. When academics teach students without having much formal knowledge of how students learn (Fry et al., 1999), these challenges tend to be accentuated, which is likely to negatively impact their capability to provide meaningful feedback to students that makes a difference to their learning and facilitates improvement. Boud and Molloy (2013) also observed feedback processes in higher education are commonly difficult to manage and carry out effectively and do not fulfill their aspiration of significantly influencing student learning.

Given teacher challenges in feedback provision, some studies offered valuable suggestions about how feedback could be more effectively provided (Nicol, 2010; Yang, M., and Carless, 2013). For instance, to explore how teachers might optimally construct dialogic feedback in order to foster students' productive learning, Yang and Carless highlighted six features of effective feedback that involve teachers: (1) stimulating student engagement with disciplinary problems through dialogic feedback; (2) developing student self-regulation through inducting students to the multiple purposes of feedback and their active role in generating, processing and using feedback; (3) nurturing collaborative and mutually trusting teacher-student and peer relationships; (4) showing sensitivity to students' emotional responses and psychological needs; (5) being flexible in the provision, timing, forms and sequencing of feedback, to facilitate student uptake; (6) mobilizing disciplinary and non-disciplinary resources for feedback provision, especially new technologies. Most recently, Carless and Winstone (2020) propose a new framework for teacher development of feedback literacy that includes three key dimensions: (1) a design dimension describing designing feedback processes that target student feedback uptake and evaluative feedback expertise development; (2) a relational dimension that focuses on the interpersonal side of student and teacher feedback exchanges; and (3) a pragmatic dimension outlining teachers' management of the compromises inherent in classroom feedback practices.

In spite of much theorizing about teacher provision of feedback, relatively fewer quantitative studies have been conducted to explore how students actually engage with feedback. Existent research suggested that culture and values of different disciplines might influence formation of feedback and feedback engagement (Winstone et al., 2020), and productive feedback processes tend to be shaped by disciplinary conventions and practices (Esterhazy, 2018). In the area of academic English studies in higher education settings, there have been small-scale qualitative investigations into students' engagement with teacher written feedback on their writing performance (e.g., Han and Hyland, 2015; Zhang and Cheng, 2018; Han and Xu, 2019; Hu, 2019). For example, Han and Xu (2019) investigated whether and how EFL students' feedback literacy changed during the course and whether and how teacher feedback on peer feedback influenced the development of the students' feedback literacy. They found that teacher feedback had effects on individual students when it was compatible with learner factors, such as English learning motivation, beliefs, and language ability. Examining aspects of English-medium master's theses that supervisors focused on in their feedback in four disciplinary areas, Neupane Bastola and Hu (2020) found that students, English studies students in particular, valued supervisors' feedback as they thought they themselves might not be able to notice and correct language problems in their work. Neupane Bastola and Hu also noted that supervisory feedback varied in different disciplines, and such feedback did not cater to students' expectations and needs. While these small-scale qualitative studies contribute to the understanding and knowledge of feedback formation and engagement in academic English contexts, the results may not be generalized to larger student populations in different pedagogical and institutional contexts. A holistic picture of teacher feedback practices, student feedback engagement, and their learning achievement is still lacking in higher education (Adams et al., 2020). The purpose of this article was, therefore, to examine the characteristics of teacher feedback practices and student feedback experience, as well their associations with learning outcomes in an English Studies course at a key and non-key university. We are interested in a comparison of teacher feedback and student feedback experience between these two kinds of universities in China because previous research suggests that institutional variance influences student learning experiences (Yin and Wang, 2016). For example, Gan et al. (2018; 2019a) found that students from prestigious universities tended to use deep learning strategies more frequently than students from less prestigious universities.

Specifically, our study is guided by the following three research questions:

1. What are the levels of teacher feedback practices, student feedback motivation and feedback behavior in a tertiary English Studies course?
2. Are there any differences in teacher feedback practices, student feedback motivation and feedback behavior in an English Studies course between a key and non-key university?
3. What are the relationships among teacher feedback practices, student feedback motivation and behavior, course satisfaction and course exam results in an English Studies course at a key and non-key university?

## Literature Review

### Teacher Feedback Practices

Feedback is recognized as having a formative effect on learning. Research on formative assessment has emphasized the importance of feedback being a vital link between teacher assessment and student learning following that assessment (Black and Wiliam, 1998; Sadler, 1998). As a result of feedback's formative effect on learning, students' knowledge and skills are formed into a more developed stage than they were prior to implementation of the particular feedback intervention (Hargreaves et al., 2000). As discussed above, viewed within the context of the constructivist theory of learning, teachers play an important role in facilitating student feedback engagement (Sadler, 1998; Carless, 2015). From a sociocultural perspective, feedback processes tend to be mediated by teacher conceptions of teaching, student relationships with their teachers, and structural constraints, such as modularized programs or large classes. Existing in a multitude of forms, such as oral responses, grades, or written comments, and embedded in an intricate mix of directives or dialogue, feedback can be given in a planned or spontaneous manner (Assessment Reform Group, 2002; Plank et al., 2014). Within the context of the traditional classroom teaching where transmission of information is the primary task of teaching, feedback tends to be one-way communication to provide information to help students to learn (Askew and Lodge, 2000). The information communicated (i.e., grades, scores, and judgmental comments) is usually *verification feedback*. This verification feedback, sometimes labeled outcome feedback, provides binary information describing whether or not results are correct (Butler and Winne, 1995). This type of verification feedback carries no additional information about the task other than its state of achievement. Hence, it is likely that verification feedback provides minimal external guidance for a learner about how to self-regulate (Butler and Winne, 1995).

Within the context of the classroom teaching that views learning as a process whereby students actively construct their knowledge through trying out and understanding, feedback, however, is more likely to help make connections and engage students in a deeper process of understanding (Askew and Lodge, 2000). This is best illustrated by the *facilitative feedback*, which “provides comments and suggestions to help guide students in their own revision and conceptualization” (Shute, 2008, p. 157). According to Shute, one widely used facilitative feedback strategy is scaffolding which allows learners to perform more advanced activities and to engage in more advanced thinking and problem solving than they could without such assistance. Such explicit support in the form of teacher scaffolds can be gradually removed when students gain their cognitive footing (Vygotsky, 1987).

Lipnevich and Smith (2009) observed that in the daily classroom, apart from verification feedback in the form of grades, praise is probably the second most common type of feedback that is often present in class situation Kanouse et al. (1981) define *praise* as positive evaluation made by a person of another's products, performances or attributes, whereas Baumeister et al. (1990) refer to praise as favorable interpersonal feedback. Teachers may generally hold the belief that praise forms an important function in motivating, rewarding, and enhancing student self-esteem (Askew and Lodge, 2000). While it is usually assumed that a feedback message containing praise enhances motivation and leads to improvement of individuals' performance (e.g., Pintrich and Schunk, 2002), some studies suggest that giving praise in a general or indiscriminate way is likely to be unhelpful (Brophy, 1981; Kluger and DeNisi, 1996; Hattie and Timperley, 2007). Hattie and Timperley (2007) argue that praise addressed to students is unlikely to be effective because it usually contains little task-related information and is rarely converted into more engagement, commitment to the learning goals, enhanced self-efficacy, or understanding about the task. Yet from a positive psychology perspective, Dweck (2008) explains that effort or “process” praise (e.g., praise for engagement, perseverance, strategies, and the like) fosters hardy motivation and keeps students focused on processes they can all engage in to learn.

Drawing on the feedback research reviewed above, the study reported in this paper operationalized three types of teacher feedback practices, i.e., *Verification feedback, Facilitative feedback*, and *Praise*, and aimed to contribute to knowledge about how teacher classroom feedback practices predict students' feedback experience and their learning outcomes in a higher education context.

### Student Feedback Experience

In line with socio-constructivist perspectives that view feedback as a social practice in which engagement is influenced by individual and contextual factors, Price et al. (2011) proposed a model of *student feedback action* encompassing several stages in the process to leading to a considered response. These stages include: *collection of teacher assessment feedback, immediate attention, cognitive response*, and *immediate or latent response*. Price et al. observed that failure to collect assessment feedback in the first stage is common although “collection” is the most visible indicator of student intention to engage. In the second stage, it was found that most students will read or listen to feedback at least once, but some students may ignore it and put the feedback in the bin. The next stage of cognitive response is considered the most critical point of engagement where students are supposed to work with assessment feedback to internalize it in relation to their learning goals, but Price et al. found that this was not a common occurrence among students. In the final stage, students' tendency to act on teacher feedback usually depends on a combination of intrinsic and extrinsic motivation. Across these stages, Price et al. posited that each stage may not be of equal importance in engagement, but can prompt further engagement or disengagement as a precursor to the next stage of the process.

While Price et al.'s (2011) conceptualization of how students act on teacher feedback contributes to understanding of students' feedback experience, a self-regulated learning (SRL) perspective should be made complementary to Price et al.'s conceptualization in explaining student feedback action. A number of models of SRL depict how learners actively generate and use feedback for themselves in the daily classroom (e.g., Butler and Winne, 1995; Nicol and Macfarlane-Dick, 2006). When students complete an academic task or an assignment, they are actively involved in monitoring and regulating their own performance, both in relation to desired task performance and in terms of the tactics used to complete the task. In this context, the students take the initiative and provide feedback to themselves which, in turn, functions as information contributing to students' regulation of subsequent cognitive engagement. In addition to the *internally generated feedback* inherent in this process of engaging in academic tasks, students also actively construct their own understanding of feedback messages derived from external sources (e.g., tutors and peer classmates) (Nicol and Macfarlane-Dick, 2006). The *external feedback information* may confirm, add to or conflict with students' interpretations of the task and the path of learning. The SRL models thus illustrate how student feedback engagement integrates with the self-regulatory processes underlying classroom academic learning activities, and provide the theoretical evidence that students themselves are able to occupy a central and active role in the feedback processes.

On the other hand, one shortcoming of the existent theorizing of student feedback experience reviewed above is that these theoretical models failed to include motivational factors explaining what drives students to act on teacher feedback or seek feedback. Dweck (1999) argue that students possess qualitatively different motivational frameworks that may affect both their responses to external feedback and their commitment to the self-regulation of learning. Particularly relevant to the present study is the Expectancy-Value Theory (EVT) which is currently considered to be an important theoretical lens to explaining individual behavioral choices and practices in relation to a given domain or a specific task (Wigfield and Eccles, 2000). From the EVT perspective, learners' subjective values are assumed to influence directly their educational and behavioral choices (Pintrich, 2004). These values concern the incentives or reasons for performing a particular activity, and typically include intrinsic value (interest) and utility value (usefulness). Drawing on these subjective values illustrated in the EVT, the present study operationalized student *feedback motivation* as consisting of interest in and perceived usefulness of classroom feedback practices.

### Teacher Feedback, Student Feedback Experience, and Learning Outcomes

Teacher feedback has been regarded as one of the most powerful influences on student learning and achievement (Sadler, 1998). Black and Wiliam's (1998) meta-analysis of 250 studies of feedback carried out in the school sector shows that effective feedback leads to learning gains, providing evidence of the value of feedback in facilitating students' learning. Nevertheless, in a meta-analysis of 131 studies on the impact of feedback on performance, Kluger and DeNisi (1996) reported that one third of the studies reviewed showed negative effects of feedback on learning, suggesting that the specific mechanisms relating feedback to learning are still mostly murky (Shute, 2008). Given the mixed effects of feedback on students' learning documented in the previous research, it is imperative that more empirical feedback research is needed to provide the evidence related to its impact on learning and achievement. Handley et al. (2011) argued that a shortcoming of previous feedback effectiveness studies was that students' active engagement as a result of multiple feedback encounters in different educational settings has been usually ignored. Recognizing the pivotal role that learners play in the feedback process, Handley et al. calls for more research into the process by which students receive, use and take action on their feedback.

Positioning feedback within a wider framework in relation to self-regulated learning, Kyaruzi et al. (2019) examined the extent to which secondary students' perceptions of their mathematics teachers' feedback practices and quality of delivery predicted their feedback use, as well as the extent to which students' perceptions of their own feedback use predicted their mathematics performance. They found that the quality of teacher feedback delivery and scaffolding positively predicted students' feedback use, whereas teacher monitoring negatively predicted feedback use. The study also found that students' feedback use had a small, statistically significant relationship to their mathematics performance. Guo (2020) explored the grade-level differences in teacher feedback, students' SRL, and their relationship patterns in the context of Chinese secondary schools. Guo noted that praise generally exhibited the strongest correlations with SRL regardless of grade level, as well as small correlations between criticism and SRL regardless of grade level. Furthermore, Guo noted a negative correlation between directive feedback and 10th graders' SRL but positive correlation between these variables among 11th and 12th graders. She also found that scaffolding and verification feedback each had a positive correlation with 11th graders' SRL.

In the higher education sector, however, relatively fewer quantitative studies have examined the linkage between feedback and student learning performance. One recent exception is that Adams et al. (2020) identified a model in which relationships between students' perceptions of feedback and their educational attainment were mediated by academic self-efficacy. Gan et al. (2020) examined Chinese university students' feedback behavior and preference in academic English course settings and their relations to English language self-efficacy. Gan et al. found that students were more likely to act on teacher feedback than to proactively seek feedback, and that English language self-efficacy had a significant influence on both feedback behavior and preference. A limitation of Gan et al.'s study was that it did not investigate interrelationships between student feedback experience and their academic performance. The current study, therefore, sets out to examine the associations between teacher feedback practices, student feedback motivation and feedback behavior, and their learning outcomes in an English Studies course context at a key and non-key university in China.

## Materials and Methods

### Research Context and Participants

The study reported in this article was set within the context of an English Studies course traditionally known as “Integrated English” taken by second-year English major students as a key compulsory course across universities in China. Of the two participating universities in the current study, one is a national key university and one is a provincial non-key university as defined by the Ministry of Education. The “Integrated English” course is typically delivered and assessed in English. While traditional and transmission-style teaching generally prevails in English Studies courses, efforts have been made in recent years to make it more student-centered in line with the “Quality Education” movement initiated by the Ministry of Education (An et al., 2021).

The participants of the present study consisted of 308 students enrolled in the “Integrated English” course from two universities as described above. There is a global trend of girls preferring to specialize in language education. Thus, there were more girls (*n* = 277, 89.9%) than boys (*n* = 31, 10.1%). The participants were aged from 18 to 29 years, with an overall mean age of 19.79 years (*SD* = 1.68). The participants were invited to complete three questionnaires related to teacher feedback practices, student feedback experience and English Studies course satisfaction. They were informed that their participation in this study was voluntary, and that they could withdraw from this research at any time if they wanted.

### Instruments

#### The Teacher Feedback Practices Questionnaire

Based on a comprehensive review of the literature as outlined earlier in this article, given the uniqueness of the tertiary English Studies course context in which the current study was situated, a teacher feedback practices questionnaire was developed for this study. The questionnaire included three subscales: (1) Verification feedback, (2) Facilitative feedback, and (3) Praise. Items in the questionnaire were adapted from Gan et al. (2019a), Gan et al. (2020) and Guo (2020). Two English Studies course teachers were invited to offer comments on the suitability of the subscales and those items that were used to measure teacher feedback practices in English Studies course settings. In light of the comments of the two teachers, the final teacher feedback practices questionnaire used in the current study comprised 13 items as follows: Verification feedback (four items, Cronbach's α = 0.83), Facilitative feedback (six items, Cronbach's α = 0.89), and Praise (three items, Cronbach's α = 0.90). *Verification feedback* is used to confirm whether an answer is correct or incorrect (Shute, 2008). In many instances, verification feedback involves a teacher simply stating “correct” or “incorrect”; *Facilitative feedback* is intended to provide successive clues or hints for guiding students to figure out problems themselves (Guo, 2020). As one of the most frequent feedback interventions in the classroom, Voerman (2014) referred to *praise* as non-specific feedback that is potentially helpful for learning because of the positive emotions it elicits and the possible creation of expansive emotional spaces. The students were required to respond to the teacher feedback items on a 7-point Likert scale (1 = Never; 7 = Always). Following the standard translation and back translation procedures (Brislin, 1970), the English version of the teacher feedback practices questionnaire was translated into Chinese to guarantee the participants' accurate understanding of the questionnaire items.

#### The Student Feedback Motivation Questionnaire

In line with the EVT theory, we developed the student feedback motivation questionnaire which consisted of two subscales: (1) Perceived usefulness (four items, Cronbach's α = 0.96); (2) Interest in feedback (three items, Cronbach's α = 0.89). *Perceived usefulness* measures the extent to which feedback helps students understand whether and where they need to improve and what needs to be done to improve; *Interest in feedback* describes the level of enjoyment in specific feedback activities. Items on these subscales were adapted from Gan (2020) and Gan et al. (2020).

#### The Student Feedback Behavior Questionnaire

Drawing on the feedback constructs articulated by Butler and Winne (1995), and Nicol and Macfarlane-Dick (2006), the student feedback behavior questionnaire used in this study contains three subscales: (1) Action on teacher feedback (four items, Cronbach's α = 0.95); (2) Internal feedback generation (four items, Cronbach's α = 0.92); (3) External feedback seeking (three items, Cronbach's α = 0.93). Items on these subscales were adapted from Gibbs and Simpson (2004), Harks et al. (2014), Yan (2016, 2018), and Gan et al. (2020). *Action on teacher feedback* denotes students' responses to teacher feedback that they received; *Internal feedback generation* is identified with students generating feedback by themselves; *External feedback seeking* denotes students actively obtaining feedback from external sources, such as teachers, peers, or parents. A 7-point rating scale, ranging from 1 (strongly disagree) to 7 (strongly agree), was used with the items on subscales *Perceived usefulness* and *Interest in feedback*. A 7-point rating scale, ranging from 1 (never) to 7 (always), was used with the items on subscales *Action on teacher feedback, Internal feedback generation*, and *External feedback seeking*. As is the case with the above two questionnaires, the English version of the student feedback behavior questionnaire was translated into Chinese to guarantee the participants' accurate understanding of the questionnaire items, after undergoing the processes of translation and back translation (Brislin, 1970).

#### The English Studies Course Satisfaction Questionnaire

English Studies course satisfaction was measured by four items adapted from Kuo et al. (2014) who measured student satisfaction with online courses (Cronbach's α for the English Studies course satisfaction scale in the current study was 0.95; sample item: “Overall, I am satisfied with this class”). The students were required to respond to the items on a 7-point Likert scale ranging from 1 (strongly disagree) to 7 (strongly agree). The English version of the English Studies course satisfaction scale was also translated into Chinese to guarantee the participants' accurate understanding of the questionnaire items, after undergoing the processes of translation and back translation (Brislin, 1970).

#### English Studies Course Exam Score

Following Hattie's (2009) suggestion that self-reported exam scores can be a reliable measure of students' learning outcomes, the participants in this study were required to report on exam scores on the English Studies course “Integrated English.” In this exam, the students were tested on their reading comprehension, vocabulary, writing, and translation. The total examination marks are 100.

### Data Analyses

Confirmatory factor analysis (CFA) using Mplus-7 was first performed to examine the factor structure and measurement properties of the teacher feedback practices, student feedback motivation and behavior, and course satisfaction questionnaires. Descriptive analyses and correlation analyses were then conducted. Next, we conducted two multivariate analyses of variance (MANOVA) to investigate potential differences between the key and non-key university in teacher feedback practices and student feedback motivation and behavior. Finally, to explore the interrelationships between teacher feedback practices, student feedback motivation and behavior, course satisfaction and course exam scores, structural equation modeling (SEM) was performed with each university's participants' data. Indirect effects testing was used to examine the extent to which student feedback motivation and behavior mediated the link between teacher feedback practices and student course satisfaction or course exam results.

## Results

### Confirmatory Factor Analyses

CFA was performed to examine the factor structure separately for the instruments measuring teacher feedback practices, students' feedback motivation and feedback behavior, as well as student course satisfaction. As shown in Table 1, with regard to the teacher feedback practices questionnaire, satisfying model fits were found with χ^2^ = 275.19, df = 56, CFI = 0.94, TLI = 0.91, SRMR = 0.05. The factor loadings of the teacher feedback practices questionnaire ranged from 0.63 to 0.90. Regarding student feedback motivation, CFA results suggested acceptable model fits with χ^2^ = 210.17, df = 26, CFI = 0.93, TLI = 0.91, SRMR = 0.07. In terms of the feedback behavior, after CFA was carried out, unsatisfactory model fits were found with χ^2^ = 355.07, df = 62, CFI = 0.92, TLI = 0.90, SRMR = 0.12. We therefore attempted to improve the model fit by addressing the item issues suggested in the modification indices. Two items were removed because the modification indices suggested that these items significantly cross-loaded on two factors, and the modified model was reevaluated. CFA results of the modified model of the three-factor feedback behavior scale showed satisfactory model fits with χ^2^ = 140.70, df = 41, CFI = 0.97, TLI = 0.96, SRMR = 0.06. The factor loadings of items of the feedback behavior questionnaire ranged from 0.86 to 0.97. With regard to student English Studies course satisfaction, the CFA results showed a good model fit: χ^2^ = 15.84, df = 2, CFI = 0.99, TLI = 0.97, SRMR = 0.01. The factor loadings of items in this scale ranged from 0.87 to 0.96.

**Table 1:** Fit indices for measurement models of the measurements.

**Measurement**	**Factors**	***x*^**2**^**	**df**	**CFI**	**TLI**	**SRMR**
Teacher feedback practices	Verification feedback	275.19	56	0.94	0.91	0.05
	Facilitative feedback					
	Praise					
Feedback motivation	Perceived usefulness	210.17	26	0.93	0.91	0.07
	Interest in feedback					
Feedback behavior	Action on teacher feedback	140.70	41	0.97	0.96	0.06
	Internal feedback generation					
	External feedback seeking					
Course satisfaction	Course satisfaction	15.84	2	0.99	0.97	0.01

### Descriptive Statistics and Correlations

Descriptive statistics for the teacher feedback practices, student feedback motivation and behavior, and course satisfaction and course exam scores as well as correlations between these factors are provided in Table 2. For teacher feedback practices variables, Facilitative feedback (*M* = 5.53, *SD* = 1.04) received the highest score, whereas Praise (*M* = 5.20, *SD* = 1.32) received the lowest. Both the two variables of feedback motivation were reported to be at a high level (i.e., perceived usefulness: *M* = 5.70, *SD* = 1.10; Interest in feedback: *M* = 5.60, *SD* = 1.08). For feedback behavior, Action on teacher feedback (*M* = 5.51, *SD* = 1.08) was reported to be the highest, followed by Internal feedback generation (*M* = 5.44, *SD* = 1.04), and External feedback seeking (*M* = 4.56, *SD* = 1.49). The students reported a generally high level of College English course satisfaction (*M* = 5.75, *SD* = 1.12).

**Table 2:** Descriptive statistics and reliabilities (*n* = 308).

	**No. of items**	***M***	***SD***	**Cronbach's α**
**Teacher feedback practices**				
Verification feedback	4	5.31	1.22	0.83
Facilitative feedback	6	5.53	1.04	0.89
Praise	3	5.20	1.32	0.90
**Feedback motivation**				
Perceived usefulness	5	5.70	1.10	0.96
Interest in feedback	3	5.60	1.08	0.89
**Feedback behavior**				
Action on teacher feedback	4	5.51	1.08	0.95
Internal feedback generation	4	5.44	1.04	0.92
External feedback seeking	3	4.56	1.49	0.93
Course satisfaction	4	5.75	1.12	0.95
Course exam scores		80.10	7.55	

As can be seen in Table 3, for students at the key university, all teacher feedback, student feedback motivation, and feedback behavior factors had significant positive correlations with course satisfaction (0.49 ≤ *r* ≤ 0.78, *p*s < 0.001). Interestingly, teacher feedback factors showed no correlations with course exam, but all feedback motivation and feedback behavior variables had positive correlations with course exam scores, although only the correlation between one of the feedback motivation variables (i.e., *Interest in feedback*) and course exam scores reached significance level (*r* = 0.18, *p* < 0.05). In the case of non-key university students, all teacher feedback, student feedback motivation feedback behavior variables also had significant positive correlations with course satisfaction (0.38 ≤ *r* ≤ 0.73, *p*s < 0.001). However, unlike key-university students, both teacher feedback and student feedback motivation and behavior variables had positive correlations with course exam scores, with *Perceived usefulness, Interest in feedback*, and *Action on teacher feedback* showing significant correlations with course exam scores (*r* = 0.21, *p* < 0.05; *r* = 0.23, *p* < 0.01; *r* = 0.22, *p* < 0.01).

**Table 3:** Zero-order correlations between teacher feedback practices, student feedback motivation and behavior, course satisfaction and course exam scores for students from the key and non-key university.

	**1**	**2**	**3**	**4**	**5**	**6**	**7**	**8**	**9**	**10**
1. Verification feedback	—	0.78^***^	0.71^***^	0.66^***^	0.57^***^	0.65^***^	0.53^***^	0.46^***^	0.63^***^	0.16
2. Facilitative feedback	0.84^***^	—	0.76^***^	0.77^***^	0.61^***^	0.71^***^	0.61^***^	0.46^***^	0.71^***^	0.15
3. Praise	0.62^***^	0.72^***^	—	0.65^***^	0.47^***^	0.64^***^	0.53^***^	0.49^***^	0.64^***^	0.12
4. Perceived usefulness	0.71^***^	0.82^***^	0.65^***^	—	0.74^***^	0.77^***^	0.60^***^	0.28^***^	0.73^***^	0.21^*^
5. Interest in feedback	0.46^***^	0.61^***^	0.54^***^	0.66^***^	—	0.66^***^	0.71^***^	0.44^***^	0.57^***^	0.23^**^
6. Action on teacher feedback	0.59^***^	0.64^***^	0.62^***^	0.65^***^	0.70^***^	—	0.68^***^	0.36^***^	0.68^***^	0.22^**^
7. Internal feedback generation	0.54^***^	0.61^***^	0.63^***^	0.65^***^	0.75^***^	0.83^***^	—	0.49^***^	0.56^***^	0.16
8. External feedback seeking	0.38^***^	0.43^***^	0.57^***^	0.38^***^	0.51^***^	0.59^***^	0.67^***^	—	0.38^***^	0.12
9. Course satisfaction	0.66^***^	0.75^***^	0.54^***^	0.78^***^	0.60^***^	0.68^***^	0.63^***^	0.49^***^	—	0.17^*^
10. Course exam scores	−0.03	0.020	−0.04	0.107	0.18^*^	0.12	0.14	0.12	0.11	—

*** *p < 0.001,*

** *p < 0.01,*

* *p < 0.05*.

*The lower half of the triangle presents the correlations between teacher feedback practices, student feedback motivation and behavior, course satisfaction and course exam scores for students from the key university. The upper half of the triangle presents the correlations between these variables for students from the non-key university*.

### Differences in Teacher Feedback Practices Between the Key and Non-key University

**Figure 1:**
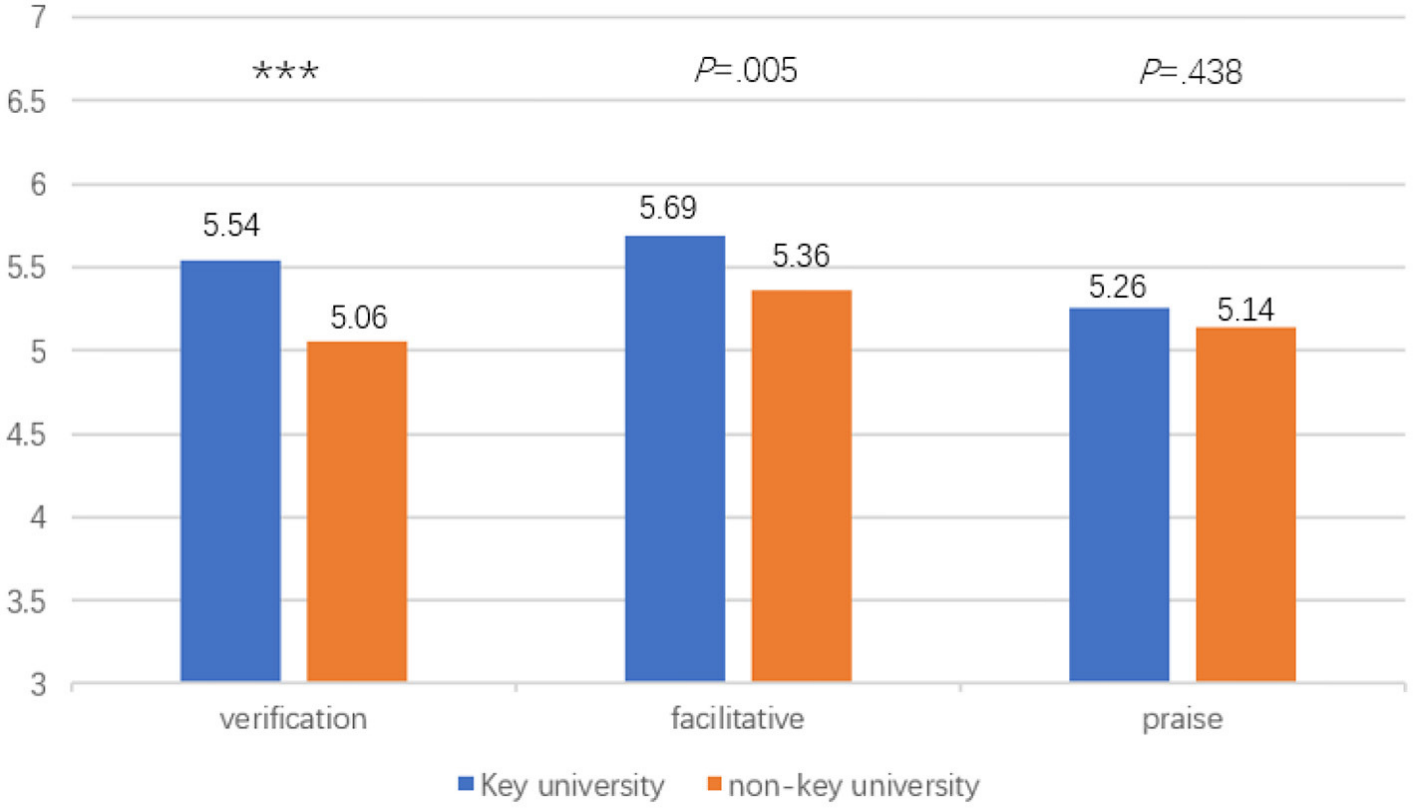
Mean differences of teacher feedback practices between students from the key and non-key university. ***p < 0.001.

MANOVA was conducted to compare the mean values of teacher feedback practices between the two universities. The results indicated that there exists a statistically significant difference between the key and non-key university on the combined teacher feedback practices variables: Wilk's λ = 0.95, *p* = 0.001, *F*_(3, 304)_ = 5.87, partial η^2^ = 0.06. When the results for three teacher feedback practices factors were considered separately, the two differences to reach statistical significance were *Verification feedback* and *Facilitative feedback*. The students from the key university reported higher mean scores on all the teacher feedback practices variables than the students from the non-key university, i.e., *Verification feedback* [*M*_(key uni.)_ = 5.54, *M*_(non−key uni.)_ = 5.06, *p* < 0.001], *Facilitative feedback* [*M*_(key uni.)_ = 5.69, *M*_(non−key uni.)_ = 5.36, *p* = 0.005], and *Praise* [*M*_(key uni.)_ = 5.26, *M*_(non−key uni.)_ = 5.14, *p* = 0.438] (see Figure 1).

### Differences in Student Feedback Motivation Between the Key and Non-key University

**Figure 2:**
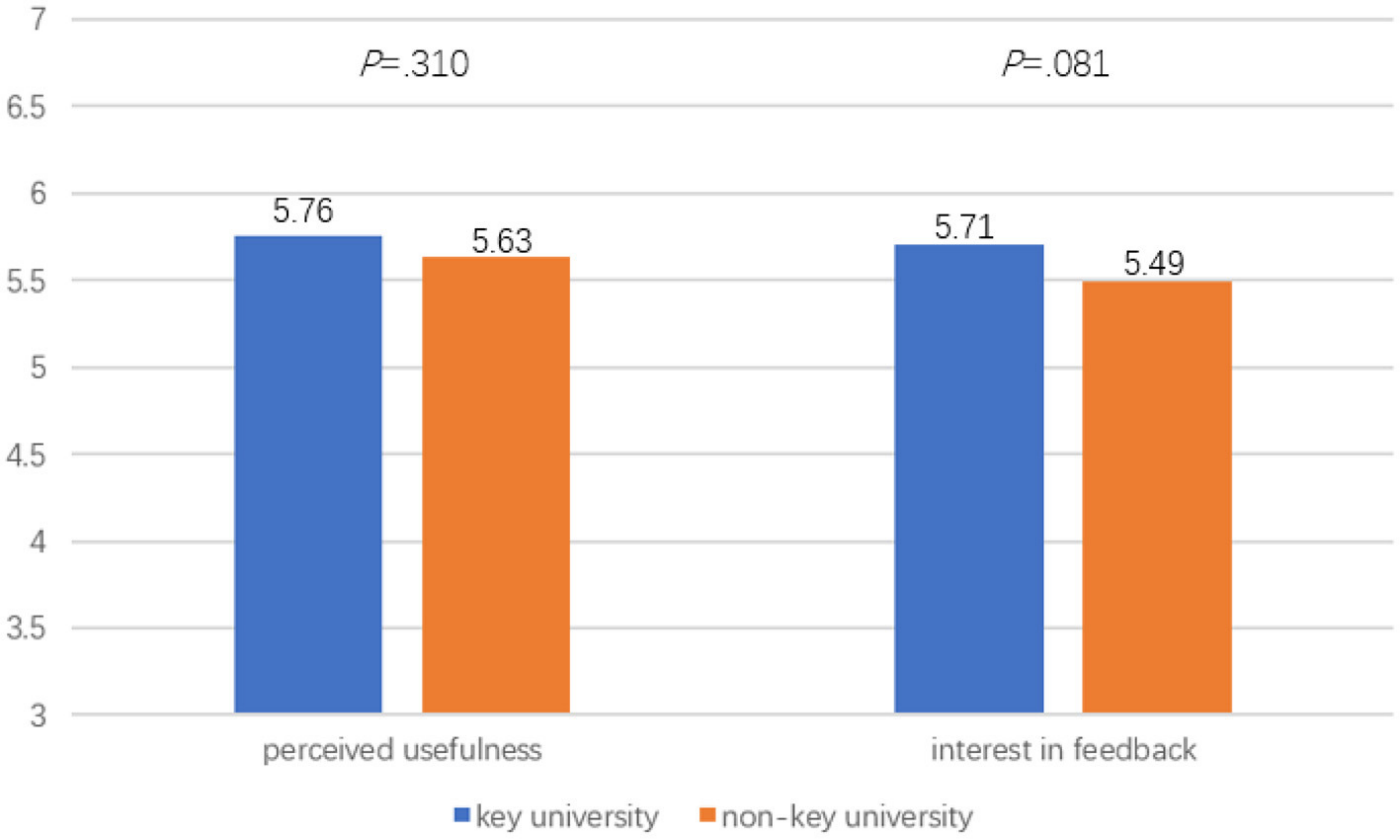
Mean differences of feedback motivation between students from the key and non-key university.

MANOVA was also performed to compare student feedback motivation between the two universities. For the effect of university type on students' feedback motivation, a non-significant main effect was noted, Wilk's λ = 0.99, *p* = 0.21, *F*_(2, 305)_ = 1.57, partial η^2^ = 0.01. While students from the key university reported higher mean scores on the two feedback motivation variables than students from the non-key university, i.e., *Perceived usefulness* [*M*_(key uni.)_ = 5.76, *M*_(non−key uni.)_ = 5.63, *p* = 0.31], *Interest in feedback* [*M*_(key uni.)_ = 5.71, *M*_(non−key uni.)_ = 5.49, *p* = 0.081], the differences did not reach significant level (see Figure 2).

### Differences in Student Feedback Behavior Between the Key and Non-key University

**Figure 3:**
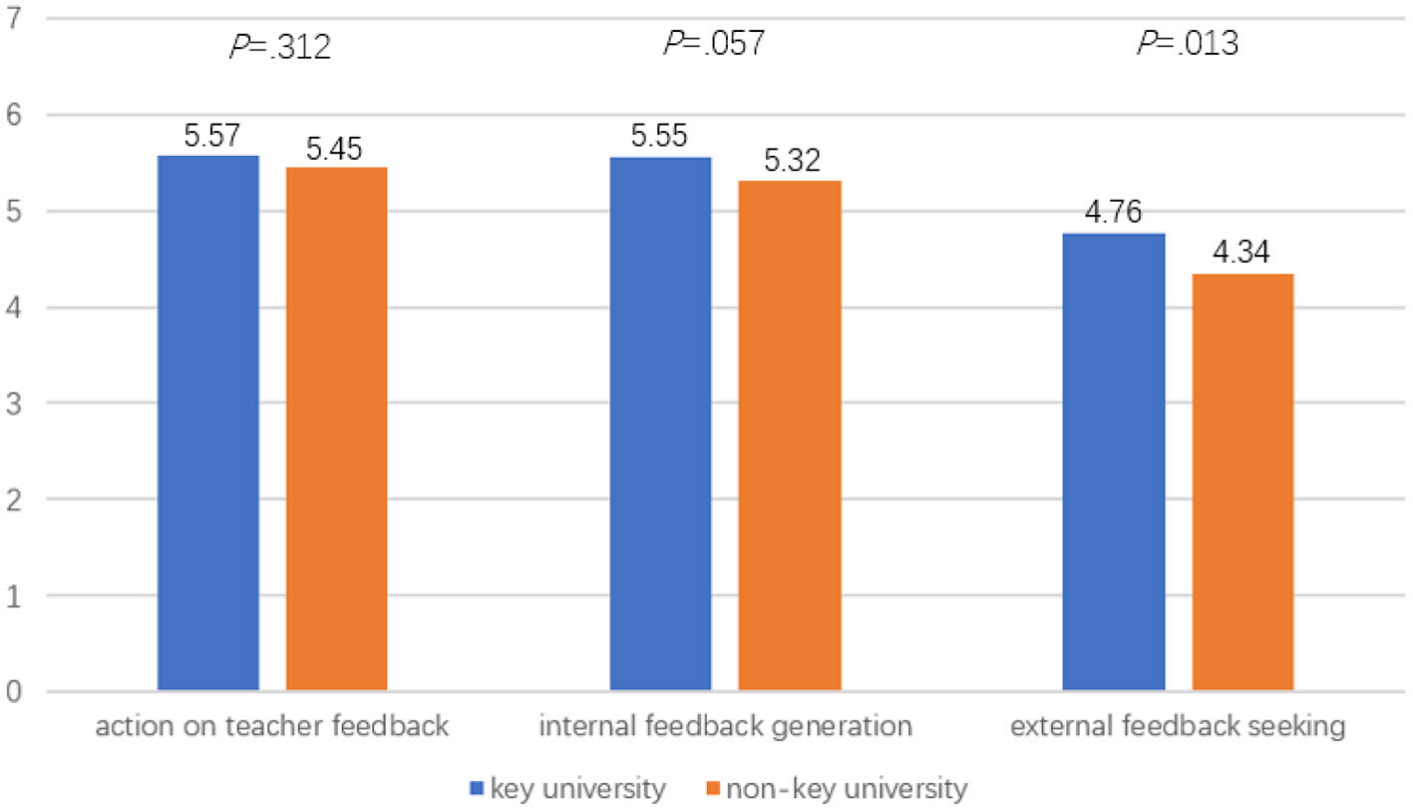
Mean differences of feedback behavior between students from key and non-key university.

Another MANOVA was performed to compare student feedback behavior between the two universities. For the effect of university type on students' feedback behavior, a non-significant main effect was noted, Wilk's λ = 0.98, *p* = 0.69, *F*_(3, 304)_ = 2.39, partial η^2^ = 0.02. While students from the key university reported higher mean scores on all the three feedback behavior variables than students from the non-key university, i.e., *Action on teacher feedback* [*M*_(key uni.)_ = 5.57, *M*_(non−key)_ = 5.45, *p* = 0.312], *Internal feedback generation* [*M*_(key uni.)_ = 5.55, *M*_(non−key uni.)_ = 5.32, *p* = 0.057], and *External feedback seeking* [*M*_(key uni.)_ = 4.76, *M*_(non−key uni)_ = 4.34, *p* = 0.013], only the difference in external feedback seeking was significant (Figure 3).

### Structural Equation Modeling

**Figure 4:**
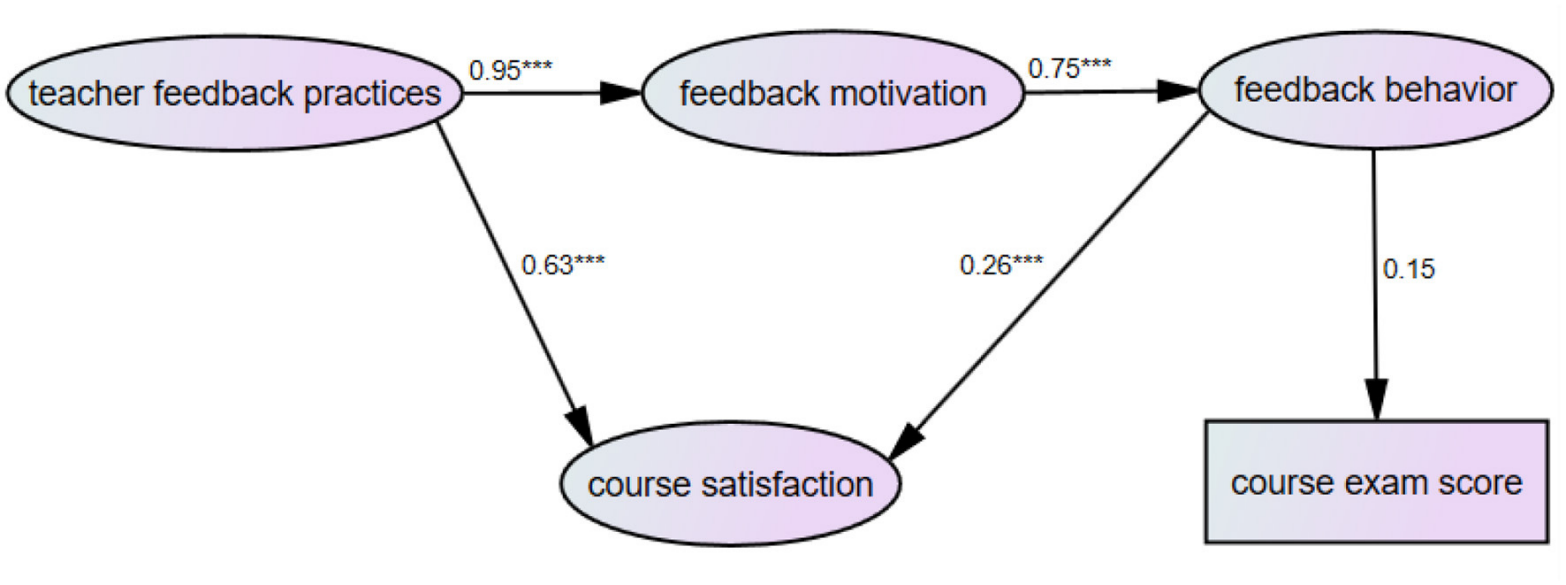
SEM model showing the relations between teacher feedback practices, feedback motivation, feedback behavior, course satisfaction, and course exam scores for students from the key university. Figures represent standardized regression coefficients. ***p < 0.001.

To investigate the relationships between teacher feedback practices, student feedback motivation and behavior, course satisfaction and course exam scores, SEM using AMOS 23 was conducted. *First, for students from the key university*, the results indicated that the model fit the data well, X^2^ = 1323.09, df = 631, *p* < 0.001, CFI = 0.91, TLI = 0.90, SRMR = 0.07. As shown in Figure 4, teacher feedback practices had significant effects on student feedback motivation (β = 0.95, *p* < 0.001) and course satisfaction (β = 0.63, *p* < 0.001). Furthermore, feedback motivation had a significant effect on feedback behavior (β = 0.75, *p* < 0.001). Feedback behavior was positively linked to course satisfaction (β = 0.26, *p* < 0.001) and course exam score (β = 0.15, *p* > 0.05), but only the link between feedback behavior and course satisfaction was significant.

The mediation by feedback motivation and feedback behavior of the effect of teacher feedback practices on course satisfaction was then tested using bootstrapping procedures. As demonstrated in Table 4, the unstandardized indirect effect of teacher feedback practices on course satisfaction via feedback motivation and feedback behavior was 0.19. The 95% bias-corrected confidence interval for the mediated effect was between 0.08 and 0.36, with a *p*-value at 0.034 for the two-tailed significance test and the standard error at 0.09. The total effect of teacher feedback practices on course satisfaction was 0.85.

**Table 4:** Unstandardized direct, indirect, and total effects for structural model.

**Predicted variable**	**Predictor variable**	**Direct effect**	**Indirect effect**	**Total effect**
Course satisfaction	Teacher feedback practices	0.65^**^	0.19^*^	0.85^**^
	Feedback motivation	0.32^*^	0.20^*^	0.20^*^
	Feedback behavior			

** *p < 0.01,*

* *p < 0.05*.

**Figure 5:**
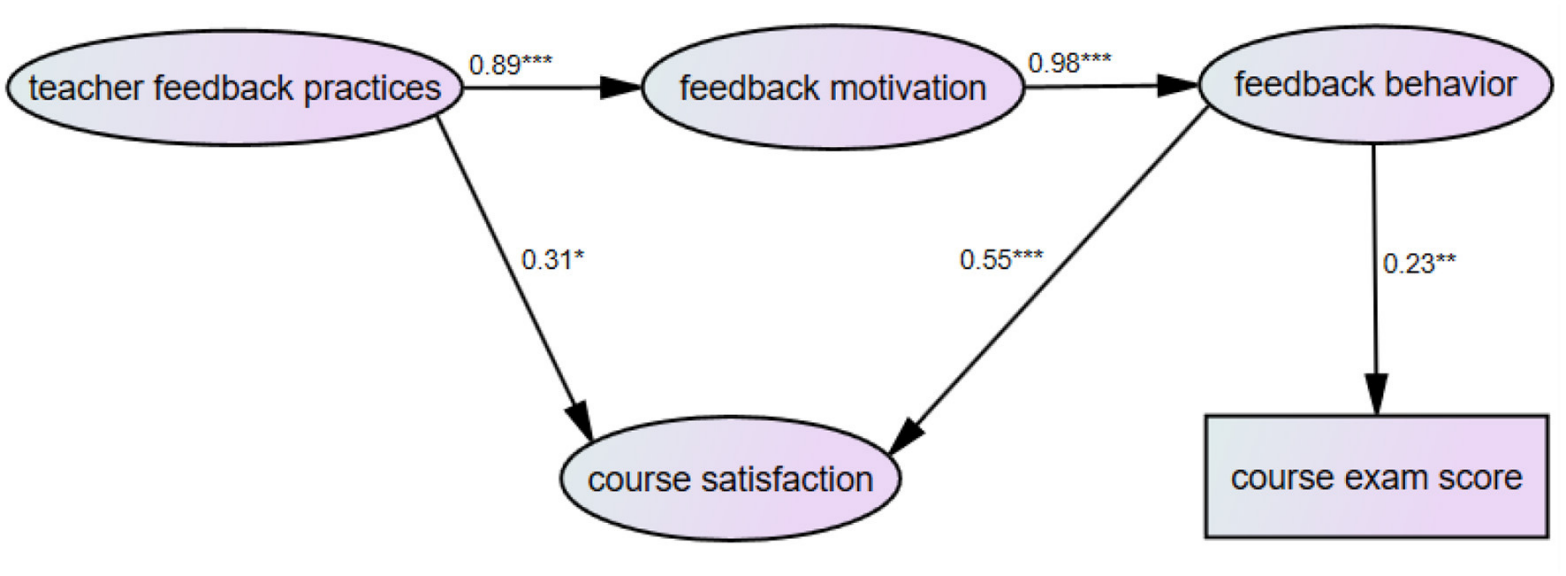
SEM model showing the relations between teacher feedback practices, feedback motivation, feedback behavior, course satisfaction, and course exam scores for students from the non-key university. Figures represent standardized regression coefficients. ***p < 0.001, **p < 0.01, *p < 0.05.

*For students from the non-key university*, SEM results also suggested that the data fit the model well: X^2^ = 1157.51, df = 633, *p* < 0.001, CFI = 0.91, TLI = 0.90, SRMR = 0.07. Teacher feedback practices were significantly associated with feedback motivation (β = 0.89, *p* < 0.001) and course satisfaction (β = 0.31, *p* < 0.05). In addition, feedback motivation was significantly linked to feedback behavior (β = 0.98, *p* < 0.001). Feedback behavior had significant effects on course satisfaction (β = 0.55, *p* < 0.001) and course exam scores (β = 0.23, *p* < 0.01) (see Figure 5).

We then tested the mediation by feedback motivation and feedback behavior of the effect of teacher feedback practices on course satisfaction and course exam results, using bootstrapping approach. As shown in Table 5, the unstandardized indirect effect of teacher feedback practices on course satisfaction was 0.49. The 95% bias-corrected confidence interval for the mediated effect was between 0.17 and 1.13, with a *p*-value at 0.009 for the two-tailed significance test and the standard error at 0.25. The total effect of teacher feedback practices on course satisfaction was 0.80. The unstandardized indirect effect of teacher feedback practices on course exam scores through student feedback motivation and behavior was 1.36. The 95% bias-corrected confidence interval for the mediated effect was between 0.34 and 2.54, with a *p*-value at 0.013 for the two-tailed significance test and the standard error at 0.57.

**Table 5:** Unstandardized direct, indirect, and total effects for structural model of non-key university.

**Predicted variable**	**Predictor variable**	**Direct effect**	**Indirect effect**	**Total effect**
Course satisfaction	Teacher feedback practices	0.31	0.49^**^	0.80^**^
	Feedback motivation	0.73^*^	0.69^**^	0.69^**^
	Feedback behavior			0.73^*^
Course exam scores	Teacher feedback practices		1.36^*^	1.36^*^
	Feedback motivation	2.02^*^	1.92^*^	1.92^*^
	Feedback behavior			2.02^*^

** *p < 0.01;*

* *p < 0.05*.

## Discussion

This study aimed to examine teacher feedback practices and student feedback experiences in an English Studies course context at a key and non-key university in China. A number of major findings need to be highlighted. First, teacher facilitative feedback was reported to be used most frequently, whereas praise was reported to be provided the least. Second, significant differences were found in teacher feedback practices (i.e., *Verification feedback* and *Facilitative feedback*) and student feedback behavior (i.e., *external feedback seeking*) between the key and non-key university in this study. Third, student feedback behavior appeared to have a stronger predictive power on student exam results than teacher feedback practices in either key or non-key university. Fourth, teacher feedback had a significant indirect effect on both course satisfaction and course exam scores via student feedback motivation and behavior in the non-key university, whereas in the key university, teacher feedback had a significant indirect effect only on course satisfaction via student feedback motivation and behavior. The present study thus contributes to a holistic understanding of the role of teacher feedback and student feedback experience rooted within an English Studies course as predictors of student course learning outcomes.

### Teacher Feedback Practices, Student Feedback Motivation, and Behavior in an English Studies Course

Teacher facilitative feedback was found to be most frequently used in both the two universities in this study, suggesting that teachers involved in the “Integrated English” course were highly inclined to provide scaffoldings to facilitate students solving problems or performing tasks independently in the daily classroom. The dominance of facilitative feedback practices in this English Studies course could be related to the recent adoption of tasked-based language teaching initiated in the current reform of the tertiary English Studies curriculum in line with a constructivist perspective of learning as opposed to traditional grammar-translation approach (Chinese Ministry of Education, 2000). In the task-based classroom, students are given more responsibility and involvement in the learning process, which is achieved through discovery learning and group work (Thornbury, 2005). Teachers using task-based approach thus tend to scaffold students forward in a productive way through discussing problems discovered at the whole-class level, or providing individual feedback to students who might have some unique difficulty in particular aspects of their group work (Gan et al., 2019b).

Note that while our study participants reported teachers using facilitative feedback most often, teachers were also perceived to use Verification feedback (*M* = 5.31) frequently. This is consistent with Guo's (2020) result that teachers generally provided verification feedback with a high frequency. According to Shute (2008), verification feedback, also known as “knowledge of results” is often intended to inform the learners about the correctness of their responses. It is generally believed that telling students that their responses were correct reinforced the cognitive processes through which the student had gone in order to arrive at the correct answer and thus would increase the possibility that the correct response would be given to a similar prompt in the future **(**Wiliam, 2013). Meanwhile, verification feedback is also often believed to enable students to correct erroneous knowledge components leading to a consequent improvement in their achievement (Harks et al., 2014), which proves to be useful in learning a second language particularly in the context where communication outside the classroom is conducted in the students' first language (Kartchava, 2019). This might explain teachers' relatively frequent use of verification feedback in the English Studies course context, which is likely to contribute to students' English language development, prompting them to notice the gap between the ideal linguistic forms and the erroneous ones and to make adjustments to their interlanguage (Lightbown and Spada, 2013).

Interestingly, although students reported teacher use of praise the least among the three types of teacher feedback practices, praise generally occurred in the “Integrated English” course with a high frequency (*M* = 5.20). In the key university, praise had significant positive correlation with course satisfaction although it had no correlation with course exam scores. In the non-key university, in addition to significant positive correlation with course satisfaction, praise had positive correlation with course exam scores, although this correlation did not reach significance level. These results suggest that praise at least impacted to some extent on student learning in the English Studies course context. From a positive psychology perspective, some researchers described positive feedback as showing support, encouragement and appreciation, or enhancing motivation, which in turn leads to improvement of individuals' performance (Pintrich and Schunk, 2002; Voerman et al., 2012). For example, Sutton (2012) suggested that when teachers conveyed appreciation of their students, the possibility of students' engagement in academically purposeful activities is enhanced. This assumption seems to be the most plausible explanation of the teachers' tendency to use praise in the English Studies course in the current study.

As discussed earlier in this article, student feedback motivation has been generally neglected in empirical research. In line with an EVT theoretical perspective (Wigfield and Eccles, 2000), this study operationalized feedback's perceived usefulness and interest in feedback as two important aspects of student feedback motivation. The participants reported a high level of both perceived usefulness (*M* = 5.70) of and interest in feedback (*M* = 5.60), which could be related to the students' preference for teacher feedback (Hu, 2019). With regard to feedback behavior, consistent with previous research (Gan et al., 2020), students in this study showed a remarkably lower level of external feedback seeking (*M* = 4.56) than action on teacher feedback (*M* = 5.51) and internal feedback generation (*M* = 5.44). Nevertheless, by measuring both feedback motivation and feedback behavior embedded within classroom processes, this study contributes to a better understanding of student feedback engagement rooted within an English Studies course.

### Differences in Teacher Feedback, Student Feedback Motivation, and Behavior Between the Key and Non-key University

Some obvious differences between the two universities in teacher feedback practices and student feedback motivation and behavior were noted. Regarding teacher feedback practices, students in the key university reported significantly more teacher use of verification feedback and facilitative feedback. There was also a more frequent use of praise in the key university, although this difference did not reach significance level. These results were consistent with Chen et al.'s (2014) finding of their study which showed that two Chinese EFL teachers' enactment of the formative assessment initiative in classrooms differed in crucial aspects, with the key-university teacher's feedback not only identifying areas for improvement, but also giving detailed and individualized suggestions on ways to improve. The English Studies course involved in this study, which is intended to provide students with comprehensive training of the skills particularly in reading, writing and translating, has been prescribed by the Ministry of Education as a core compulsory course across universities (Chinese Ministry of Education, 2000). The course has been undergoing reforms and innovation in recent years to focus on the development of students' communicative competence in line with the communicative language teaching movement. On the other hand, different universities could differ in the provision and utilization of resources, and the learning climate and intellectual environment of the whole institution (Griffin et al., 2003). Given these challenges, it is likely that teachers in the key university in the present study were better trained, which might result in a higher level of feedback activities (Gan et al., 2019a,b).

In this study, students in the key university also tended to score higher on all feedback motivation and behavior factors, and significantly higher on external feedback seeking in particular. It could be that students in the key university were more motivated and well-organized in learning, tended to perceive teachers' feedback as being useful to a greater extent, and thus involved themselves more actively in self-generating feedback and external feedback seeking than students in the non-key university. These results provide us with a more nuanced understanding of the impact of institutional contexts regarding teacher feedback practices and student feedback engagement in the classroom. The results led us to concur with Yin and Wang (2016) who argue that students' learning motivation and engagement emanate from the goals and norms presented in their institutional contexts, and that institutional characteristics impact on student psychological investment in and effort directed toward learning.

### Relationships Among Teacher Feedback Practices, Student Feedback Motivation and Behavior, and Course Satisfaction and Course Exam Results

This study represents the first attempt to empirically explore the intertwined relationship between teacher feedback practices, student feedback motivation and behavior, and course learning outcomes in a Chinese higher education setting. SEM analyses showed that teacher feedback practices strongly predicted student feedback motivation and course satisfaction in both the key and non-key university. These results are aligned with those of previous studies that reported significant predictive effects of teacher feedback on students' perceived feedback usefulness (e.g., Harks et al., 2014), feedback use (Kyaruzi et al., 2019), and students' intrinsic motivation in learning (Azevedo and Hadwin, 2005). In addition, teacher feedback practices had a significant indirect influence on course exam scores via feedback motivation and behavior in the non-key university. These results provide empirical evidence to the argument that the value of feedback is to support students in developing their ability to monitor and regulate their learning (Nicol and Macfarlane-Dick, 2006; Carless, 2015).

SEM analyses also suggested that student feedback behavior had a positive effect on both course satisfaction and course exam results particularly in the non-key university. This result reinforces the position that teacher feedback delivery alone does not lead to student learning improvement (Nicol, 2010). The result enables us to conclude that the efficacy of feedback in higher education depends on the extent to which feedback is viewed as a process, and the extent to which students are committed to this process as active users of feedback. In other words, teacher feedback can have no impact on student learning unless students engage with it (Handley et al., 2011).

Interestingly, students in this study showed a high level of course satisfaction, and correlation analyses further showed that course satisfaction was positively related to course exam scores. However, course satisfaction had no significant direct influence on student course exam scores in the SEM. This result appears to contradict some previous research studies that suggested a positive relationship between course satisfaction and students' level of learning in online learning context (Kizil, 2020). The lack of significant influence of course satisfaction on course exam scores in the SEM in the current study might be due to the controlling for other variables in the model. When course satisfaction and other variables were analyzed together in the model, the correlation decreased. It could also be that while students participating in this particular English Studies course showed a generally high level of course satisfaction, satisfaction appeared not adequate in prompting students to invest effort directed toward learning. As such, more future studies are needed to explore the potential discrepancy between course satisfaction and course exam results.

## Implications

This study confirms the strong positive impact of teacher feedback practices on student feedback motivation and course satisfaction, and underscores the crucial role of student feedback behavior in student learning outcomes. Future research can examine the extent to which different types of teacher feedback practices effect student feedback engagement. Similarly, further research is needed to explore how each dimension of feedback motivation and behavior operationalized in this study impacts on student learning outcomes. Hattie and Timperley (2007) highlight that when feedback is directed appropriately, it can assist students to comprehend, engage, or develop effective strategies to process the information intended to be learned. Since our study demonstrates that teacher feedback practices have important direct influence on student feedback motivation and course satisfaction, a practical implication is that there is a need to promote and discuss teachers' awareness of the power of feedback in initial and in-service teacher training (Vattøy and Smith, 2019). Such teacher training should help to raise teachers' consciousness with regard to the creation of conditions that provide feedback opportunities, and facilitate communication with students about the nature of productive feedback processes and their role as active feedback users (Carless, 2015). This may involve teachers rescinding their traditional authority as subject experts in order to understand and address the individual needs of each student and build up a relationship of trust with students. Such a relationship of trust with students is essential to giving and receiving of feedback as students tend to feel comfortable with teachers who are approachable and open to alternative interpretations of assignment questions (Sadler, 1998), and most importantly who care about their students (Sutton, 2012).

The central role of student feedback behavior in students' learning outcomes further suggests that students need to receive in-class training that helps them to be aware of the pivotal role that they play in learning, and experience the value of being active partners in assessment and feedback processes (Carless and Winstone, 2020). Teachers thus need to be charged with the responsibility for creating an environment that affords students opportunities to generate information about, monitor, regulate and attend to the quality of their academic work (Hawe and Dixon, 2016). This necessitates teachers capable of understanding of the theories of self-regulation and addressing how self-regulation can practically support students' learning improvement in the classroom (Panadero, 2017).

## Conclusion and Limitation

This study filled the research gap by presenting a holistic picture of teacher feedback practices and student feedback experience in an English Studies course context, and by examining how teacher feedback practices and student feedback experience influence student learning outcomes (i.e., course satisfaction and course exam scores). The results of this study provide empirical evidence to the argument that the relationship between teacher feedback and learning achievement is necessarily mediated by the more proximal factor of learner engagement with feedback (Winstone et al., 2017). Despite its contribution to the literature, some limitations need to be acknowledged. First, as the participants in this study were all second-year students attending a similar English Studies course from two universities, representativeness of the research sample might be limited. Future studies should include students from more diverse courses in different educational institutions so that findings of the current study can be properly tested. Second, as the current study adopted a cross-sectional design, the SEM results only indicate associations between variables. It is not possible to prove the causal relations established among the various constructs. Future research that adopts a longitudinal or experimental design is needed to establish causal claims.

## Data Availability Statement

The raw data supporting the conclusions of this article will be made available by the authors, without undue reservation.

## Ethics Statement

The studies involving human participants were reviewed and approved by University of Macau Ethics Assessment Committee. The patients/participants provided their written informed consent to participate in this study.

## Author Contributions

ZG conceived the idea and developed the materials. ZA carried out the data collection. ZG and ZA took the lead in writing the manuscript. ZG, ZA, and FL provided the critical feedback. All authors read and approved the final manuscript.

## Conflict of Interest

The authors declare that the research was conducted in the absence of any commercial or financial relationships that could be construed as a potential conflict of interest.
